# Full Zirconia Implant-Born Prosthetic Rehabilitation with CAD/CAM Technology after Accurate Digital Planning. A Case Report

**DOI:** 10.3390/ijerph18157998

**Published:** 2021-07-28

**Authors:** Riccardo Scaringi, Michele Nannelli, Alessio Franchina, Giuseppe Lizio, Luigi V. Stefanelli, Michele Pagliarulo, Francesca De Angelis, Gerardo Pellegrino

**Affiliations:** 1 Private Practice, Via Del Futurismo, 8, 20138 Milano, Italy; rs@riccardoscaringi.com; 2 Private Practice, Via di Caciolle, 2/H, 50127 Firenze, Italy; nannellim@inwind.it; 3 Private Practice, Periodontal and Dental Implant Surgery, 36100 Vicenza, Italy; alessiofranchina@icloud.com; 4 Oral Surgery Unit, Department of Biomedical and Neuromotor Sciences, University of Bologna, Via San Vitale 59, 40125 Bologna, Italy; 5 Private Practice, Periodontal and Dental Implant Surgery, 00145 Roma, Italy; gigistef@libero.it; 6 Faculty of Dental Medicine, University of Plovdiv, 4000 Plovdiv, Bulgaria; michele.pagliarulo2000@gmail.com; 7 Department of Oral and Maxillo-Facial Sciences, Sapienza University of Rome, 00185 Rome, Italy; francesca.deangelis@uniroma1.it; 8 Researcher Oral Surgery Unit, Department of Biomedical and Neuromotor Sciences, University of Bologna, Via San Vitale 59, 40125 Bologna, Italy; gerardo.pellegrino2@unibo.it

**Keywords:** Zirconia rehabilitation, CAD/CAM technology, dental implantology

## Abstract

CAD/CAM technology can enhance the dentistry application of ceramic materials that meet the more relevant biocompatibility and aesthetics demands. In implant-borne prosthesis rehabilitation, yttria-stabilized zirconia appeared to be a valid alternative to metal-alloys and titanium, with comparable mechanical properties and even better interaction with bone and soft tissues. The improvement of monolithic CAD/CAM manufacturing allows for a reliable, predictable, and rapid workflow that can correspond to a holistic treatment philosophy associated with zirconia fixtures. This reported clinical case highlights the advantages of this approach in resolving particularly functionally and aesthetically complex situations. A 40-year-old patient with permanent canine impaction and the persistence of a deciduous tooth compromised by caries was successfully rehabilitated with the surgical removal of the enclosed tooth, the seating of a mono-phase zirconia implant after the deciduous extraction and its loading with a zirconia single crown, without any clinical or radiographical alteration up to seven years follow-up.

## 1. Introduction

The aesthetic demands of implant-borne prosthetic rehabilitation of a mono-edentulism in the frontal zone are challenging, and often necessitate the involvement of the proximal teeth with fixed dental bridge solutions to compensate hard and soft tissue alterations [1]. The implant approach requires careful prosthetic and surgical planning, and sometimes includes a surgical and orthodontic pre-prosthetic intervention for optimizing the implant positioning and functional loading [2,3]. Digital technology provides substantial help in designing and pre-visualizing the intended outcomes making patients more conscious about treatment steps and final results. This technological support has improved the accuracy of the planning phase and the precision of the restorations, and has led to higher aesthetic quality [4].

Digital Imaging and Communication in Medicine (DICOM) data on cone-beam tomography bone status, and 3D standard tessellation (STL) data on the topographical and superficial features of the teeth and soft tissues from an intraoral scanner (IOS), are paired using dedicated software, allowing the correct implant-prosthetic rehabilitation to be carried out according to anatomical and prosthetic demands [5].

Computer-aided design (CAD)/computer-aided manufacturing (CAM) technology was first adopted in restorative dentistry in 1985 [1], and since then, an increasing number of chairside systems have moved from virtual design to a computer-assisted rapid prototyping, milling, or growing option. This has enabled faster treatment and has reduced the errors associated with different phases of production, with a more precise marginal and internal fit of the prosthesis [2,3,6,7]. The CAD/CAM systems needed an adequate material to be modeled, and full-ceramic prosthetic crowns and bridges became an alternative to metal-ceramic crowns [8].

These materials evolved from a fragile feldspathic crystalline structure to zirconia oxide ceramics, with a dense monocrystalline homogeneity. Zirconia was revealed to have high flexural strength values [9]. Subsequently, yttria-stabilized zirconia polycrystal (Y-TZP) was developed as a core material to reduce the risk of bulk fractures. As a result, the range of applications was enlarged from single crowns and short-span fixed dental bridges, to multiunit and full-arch zirconia frameworks through the CAD/CAM milling procedure of sintered blocks [7,10].

Despite the more customized restorative structures able to be obtained through the use of digital technology, the use of ceramic materials was reevaluated after doubts arose over the bio-inertia of titanium with possible hypersensitivity [11]. Furthermore, higher concentrations of metal corrosion products were correlated with the duration of follow-ups in titanium implant-borne rehabilitations, suggesting that these molecules may play a role in causing bone resorption and loss of osseointegration [12]. Indeed, ceramic fixtures were proposed a long time before the development of informatics [13,14], and were rediscovered after zirconia demonstrated physical properties comparable to titanium and metal-alloys. The level of osseointegration of zirconia implants is the same as titanium implants with well-documented rough surfaces. A significant reduction in biofilm formation on ceramic surfaces was reported with a lower plaque formation [15]. Good results in terms of implant success were reported for up to approximately seven years of follow-up.

The major aesthetic property of ceramic materials compared to metal is the most important reason for the interest in zirconia, along with reported major biocompatibility. In the case of a thin gingival biotype and a complex anatomical situation, the dark shadow of titanium may be visible through the peri-implant tissues, particularly in the case of soft tissue retraction over time [2].

This paper reports on a complex case of anterior zone mono-edentulism, managed with computerized technology for the planning phase and the prosthetic superstructure realization, with both fixture and crown in zirconia.

## 2. Materials and Methods

### 2.1. Planning

A forty-year-old non-smoker patient in good health (ASA 1) with a persistent deciduous canine and bone-impacted permanent canine was referred to our dental office for a definitive aesthetic and functional rehabilitation.

**Figure 1:**
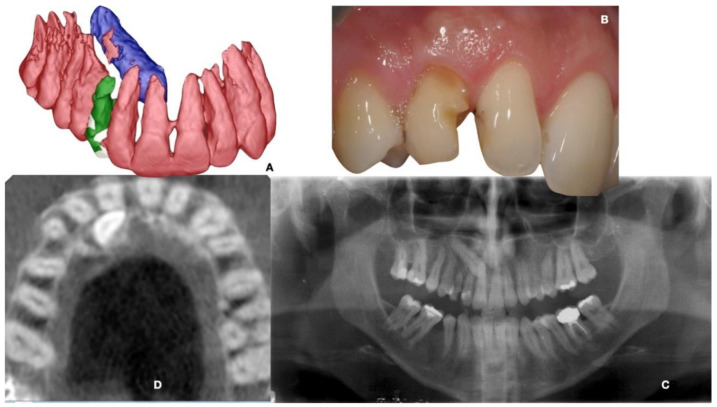
Frontal 3-dimensional (3D) view of the segmented teeth showing the palatal position of the impacted canine related to the other teeth (**A**); Clinical status with the loss of enamel tissue due to the decay process (**B**); Opt x-ray (**C**) and axial slice of CBCT confirming the palatal position of the crown of the impacted canine and its relation with root of teeth 1.1 and 1.2 (**D**).

**Figure 2:**
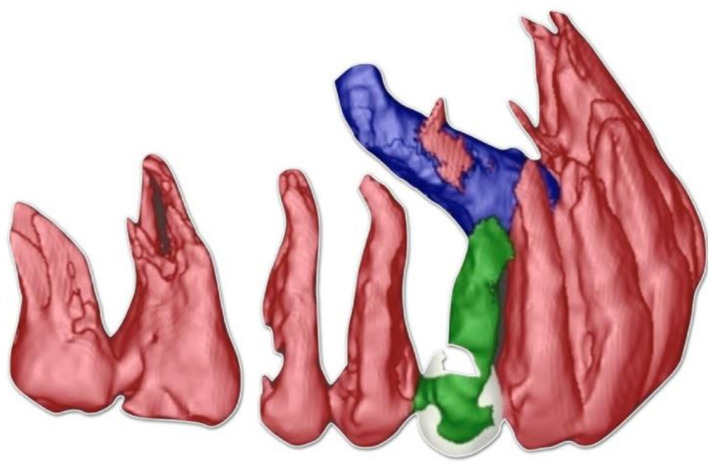
Lateral 3D view of the segmented teeth.

Figure 1 and Figure 2 show the clinical, radiological and 3D-reconstructed status at the baseline.

Different therapeutic options were proposed (summarized in Table 1).

It was useful to make a diagnostic examination to evaluate the canine’s exact position, and the residual space between the elements adjacent to the one of interest. The deciduous element was affected by caries and was slightly mobile, with a poor prognosis. To plan for an extraction of the impacted canine which retained the integrity of the proximal teeth, and spared the bone tissue as much as possible, a CBCT was taken and a segmentation of the teeth was carried out. The CBCT machine was a MyRay-SkyView Viewer Manager 1.2.0.6 Dental (operative conditions: FoV 9″/X-ray exposure time: 16.2 s/typical effective dose: 49.0 uSv/bone-high resolution). Since the patient insisted on not using a metallic implant, we opted for a monotype ceramic fixture (Ceramic Implant Monotype 4.1 × 12 mm, Straumann Implant System, Basel, Switzerland).

**Figure 3:**
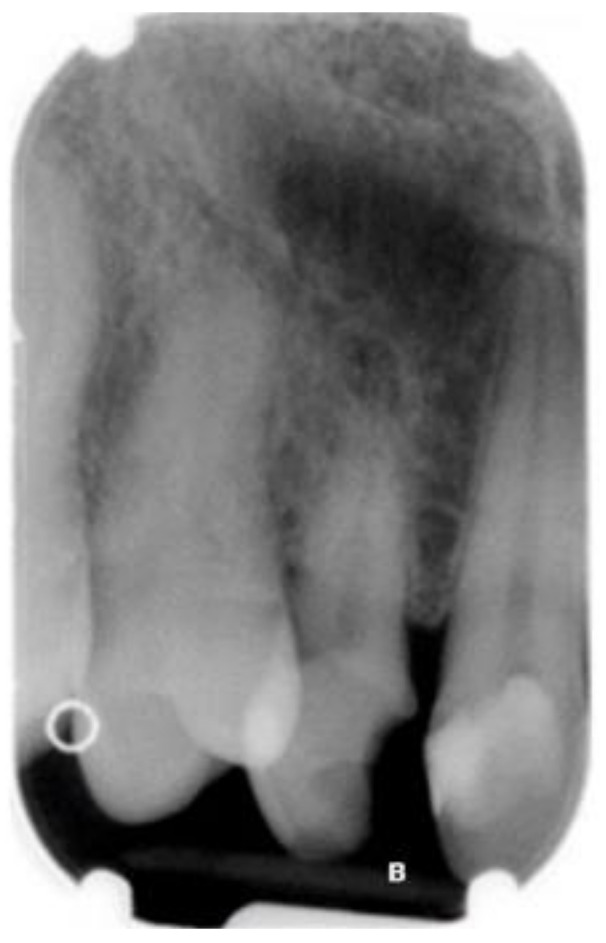
Endoral X-ray performed four months after cuspid extraction to assess the bone healing.

The canine surgical extraction was performed under regional anesthesia using a piezoelectric instrument to reduce bone removal and the risk of damaging the roots of the neighboring teeth. After 4 months of healing, the bone site was assessed again with intraoral radiographs (Figure 3).

### 2.2. Surgical and Prosthetic Procedure

The various formats are interpreted by the software and processed in such a way to be transferred to the CAD/CAM system. A preliminary impression of the arches was taken using an intraoral scanner (IOS). The adopted scanner was iTero (Align Technology Inc., 2820 Orchard Parkway, San Jose, CA 95134, USA). The iTero uses a red laser beam and parallel confocal imaging technology to capture up to 100,000 points of laser light with an accuracy of <20 micron. The STL files obtained with IOS were used to make a wax-up that was necessary to evaluate the correct morphology, dimension, and exact position of the prosthetic crown, as the occlusal space between the adjacent teeth had been reduced. For this analysis, the radiological DICOM files from the CBCT, the STL files from the IOS, and the laboratory scans were paired in a software platform, allowing the rehabilitation to be planned according to the anatomic and prosthetic demands.

After the completion of the bone healing, the extraction of the deciduous element and the implant placement was carried out in the same surgical setting. It was not a real immediate post-extraction implant insertion as the resorption of the roots allowed the bone to completely fill the alveolar space. Particular attention was paid to the implant position, as it could not be corrected in the prosthetic portion axis, due to being a monotype implant.

**Figure 4:**
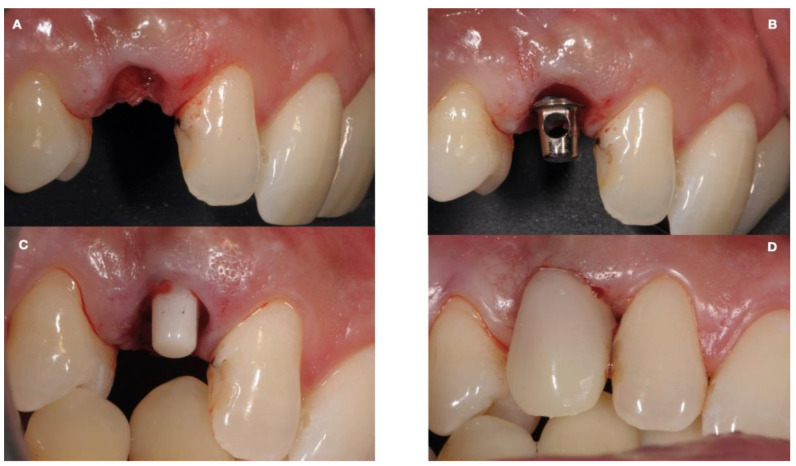
The deciduous atraumatic tooth extraction performed leaving intact hard and soft tissues (**A**); Monotype titanium implant as a guide (**B**); Monotype zirconia implant placed with a flapless approach (**C**); The immediate provisional restoration delivered at the end of the surgery (**D**).

After the pilot drill passage, the use of a dedicated abutment confirmed the correct orientation of the osteotomy, with an adequate abutment height of 4 mm. The implant insertion torque of 65 N/cm allowed the application of a temporary aesthetic crown (ION, 3M^tm^, San Paul, MN, USA) without occlusal contacts (Figure 4).

**Figure 5:**
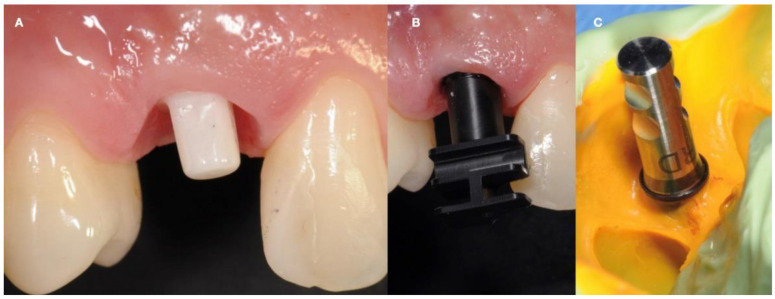
The soft tissue after a three-month healing time (**A**); A single use coping transfer to take the final impression of definitive crown (**B**); A laboratory implant analog was used to pour the master cast (**C**).

After 3 months, the provisional crown was removed and a conventional impression was taken according to the closed-tray technique, using a silicone material (Zhermack, Badia Polesine, Rho, Italy). The silicone material has a light-heavy body suitable for taking the impression of implants to better stabilize the polyether-ether-ketone (PEEK) snap-on transfer and reduce mucosal compression, resulting in a more accurate impression of the gingival area (Figure 5). Particular attention was also paid to temporary cementation to affect the peri-implant soft tissues.

**Figure 6:**
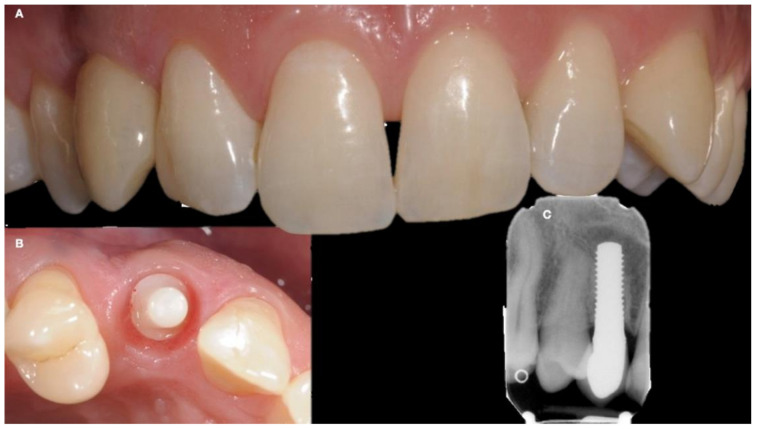
Definitive crown cementation (**A**,**B**); An endoral X-ray confirms the ideal integration of the implant (**C**).

Finally, the definitive crown in ceramic-coated zirconia was cemented with a special resinous cement, and an intra-oral X-ray was taken to confirm the prosthetic fitting (Figure 6).

## 3. Results

The treatment objectives were achieved with a good occlusion and improved aesthetics. The patient declared that they were completely satisfied.

**Figure 7:**
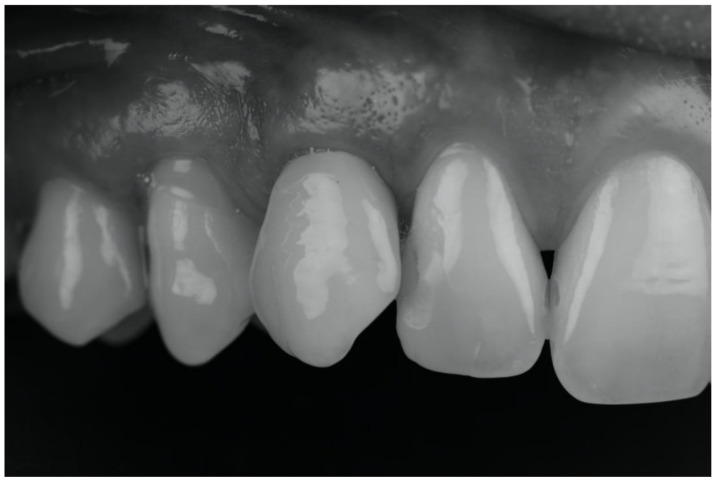
The monochromatic picture enhances the texture of both the soft tissue and the enamel surface of the crown.

Seven years after the procedure, no clinical or radiographic variation was recorded apart from minimal wear of the cervical implant portion due to an incorrect method of brushing (Figure 7). No implant or crown fracture occurred.

## 4. Discussion

This paper aims to underline the usefulness of ceramic materials, particularly zirconia, in optimizing the treatment of a difficult aesthetically demanding implant-prosthetic rehabilitation.

Zirconia is obtained through the enrichment of silicate of zirconium (ZrSiO_4_) present in fossil sands, with minerals such as yttrium, manganese, and alumina. Three different types of zirconium oxide ceramic are currently available on the market: yttrium ion stabilized tetragonal zirconia (3Y-TZP), partially stabilized magnesium zirconia (Mg-PSZ) and tempered zirconia with alumina (ZTA) [6,16]. The cited mineral molecules stabilize the crystalline structure, and confer high flexural strength (900–1200 MPa), high fracture toughness (9 to 10 MPa), and a good resistance to wear and corrosion to this white-opaque material. These properties, associated with low thermal conductivity, good biocompatibility [17], and a low affinity to bacterial plaque [18], make zirconia a superior alternative to metal–ceramic restorations in aesthetic areas. However, a few defects of this product must be noted. In particular, zirconia is prone to degradation at low temperatures. It will show a progressive surface roughness with a slow but progressive tendency to deteriorate [2].

The great advantage of ceramics is that they are easily workable with the current CAD/CAM milling or sintering systems, thus substantially reducing treatment timing, costs, and operative errors. The ability to directly realize the prosthetic components in a digitally designed project allows the clinician to better manage the entire treatment up to the finalization, with more precise and controlled outcomes. The digital method demonstrated better accuracy than the analog one, when compared to the original wax-up traditional molded and milled mock-ups for restorative treatment with porcelain veneers [4].

There is little evidence for the success of zirconia monolithic crowns. A few studies showed a 100% survival rate after 36-68 months without fractures, cracks, or chipping [19,20].

In implant-borne prosthetic rehabilitation, metal-free zirconia crowns or bridges imply a cemented connection, and a more versatile solution in the aesthetic area, with the drawback of the impossibility of removing the prosthetic superstructure for hygiene or for repair in case of structural damage.

Zirconia implants started being used as an alternative to titanium upon more frequent requirements from patients of metal-free bio-devices, based on concerns about possible immune-reactions or metal toxicity. In fact, the prevalence of titanium allergy was estimated at 0.6% in a study using the memory lymphocyte immune-stimulation assay (MELISA) [21]. Operatively, the use of a white-fixture can limit the unaesthetic consequences of a soft-tissue recession with implant neck exposure.

Nevertheless, the first ceramic implants were in alumina oxide and, over time, proved to be too fragile [14]. Hence their use, proposed since 1963, was not rediscovered with zirconia in the 1990s.

In regard to the osseointegration of zirconia, no difference was reported with titanium in terms of bone-to-implant contact (BIC). In one study, major cell-to-implant contact with multinucleated giant cells was observed with zirconia implants (17.5%) compared to titanium ones (3.9%) at four weeks [22], without any evidence of foreign body reaction. These results were interpreted as a local cellular phenomenon with no effect on the newly formed bone. Various experimental works on animal models demonstrated that zirconia is a material with strong osteo-conductivity. Oum’harmed et al. [12] compared osteoblasts’ behavior in contact with the two most used ceramics, alumina and zirconia, and highlighted the osteoconductive virtues of zirconia as it showed no cytotoxicity effects and, on the contrary, better supported the turn-over and production of the extracellular matrix. On the other hand, animal investigations showed that failed titanium oral implants might result from substantial titanium release, which can be detected as particles in macrophages found attached to failed implants.

Another aforementioned important positive property of zirconia is its non-retention of bacterial plaque, with a less marked bacterial colonization demonstrated by reduced concentrations of the metabolism products at the neck of the zirconia implant.

Generally, a ceramic implant’s bed preparation requires that the producer’s directions are strictly followed to mechanically stress the structure of the fixture [2,3,23]. Moreover, seating a monotype implant implies more attention and rigorous pre-operatory planning, since it is impossible to change the prosthetic axis with an angulated abutment. This is more relevant in situations with important spatial limitations, as are hereby reported. Indeed, the present case showed a limited interarch distance that was further reduced by the interference of the cusp of the axially rotated antagonist tooth, and a minimal mesiodistal space related to the persistence of the deciduous canine available both for the intra and extra-osseous implant portion. Realizing the prosthetic crown with a CAD/CAM system helped the clinician customize the treatment to the cases demands.

Another problem with this type of implant is the necessity of preventing the extra-osseous portion from micro-traumas that can compromise the initial phase of osseointegration, with the risk of early failure. Accuracy when choosing the adequate height of the pillar is mandatory. The role of traditionally managed provisional prosthesis should not be neglected. The temporary crown, adequately out of occlusal contacts, prevents the one-piece implant from micromotions, maintaining an aesthetic function, and allows a progressive remodeling of the peri-implant soft tissues with an easy removal for clinical examinations [24].

A further difficulty with these implants is controlling the cement flowing between the implant neck and the soft tissues, which is possibly responsible for mucositis and periimplantitis. The application of retractor wires in the cementation phase is another critical passage that complicates the procedure. Finally, in the case of pluri-edentulism with a fixed dental bridge, the necessity of an extremely precise implant seating is related to the parallelism to be obtained by the pillars.

Recently, a 5 year follow-up study with these types of implants reported a 98.4% survival rate since implant insertion, with one implant lost out of 71 placed, an overall mean marginal bone loss of 0.7 mm without statistically significant bone resorption after loading, and a stable condition of the peri-implant mucosa. These data are consistent with what is observed in the literature around titanium fixtures, with a 97.2% survival rate for implants supporting SCs [25] and 95.6% survival rate for implants supporting fixed dental prostheses [26] with up to 0.8 mm bone loss [27]. In contrast, a prospective study investigating 35 one-piece Y-TZP implants in 13 patients reported a mean marginal bone loss of 1.6 mm after 48 months [28]. Studies with an observation period longer than 5 years described a mean MBL around one-piece zirconia implants of 1.0 mm after 71.3 months [29], and 1.2 mm after 93.6 months [3], with an overall number of 154 implants investigated.

The risk of fracture in connection surfaces and screws or de-cementations of extra-osseous components, such as the abutments, marked the mono-phasic more than two-piece zirconia implants. Indeed, a three-year prospective RCT study observed worse results with zirconia CAD/CAM abutments compared to titanium ones, with an 82.2% success rate versus 100%, due to fracture occurrence at the abutment connections. For all with an internal connection, the failed abutments supported restorations in posterior areas, except for one in cuspid location [30]. In two clinical studies [3,31], zirconia abutments were cemented on implants using dual-cure resin cement. In two other clinical studies [32,33], a modifiable glass-fiber abutment was adhesively fixed to the fixture. Becker et al. [32] reported the fracture of a glass-fiber abutment 23 months after loading, and Cionca et al. [3] reported two fractured abutments in two patients at ten days and eight months. The good long-term result reported in the present paper could be attributed to the use of a one-piece implant given the surgical and prosthetic difficulties of such a device. The digital protocol was not applied for the manufacturing of the implant-abutment device, though with the awareness of the problems mentioned above, the possibility of realizing a one-piece CAD/CAM implant should not be excluded in the future.

At present, only a few ceramic systems offer two-piece implants.

Focusing on the prosthetic super-structure problems only, the monolithic approach adopted in this case report could prevent the chipping and roughening of the veneering ceramic that affected some results with zirconia recorded in the literature [34]. Another conceivable point is the obtainment and time taken for a good marginal closure with ceramics rather than with metal. A digital approach can obtain the same results with zirconia thanks to a CAD/CAM approach, starting by using the intra-oral scanner. Ferrini et al. reported better results in terms of marginal fit using IOS than with the analog protocol, maintaining the marginal gap value under 120 microns [35].

The positive results that this case presented at a long-term follow-up were the result of lengthy experience with the use of zirconia, associated with a high-level of digital technology knowledge. Hence, the possibility to treat complex cases with high aesthetic implications must be carefully evaluated and limited to selected situations. The improving technology, both for the materials and their manipulation with CAD/CAM systems, has the potential in a proximal future to fully satisfy patient requests for a more bio-compatible and aesthetic dental rehabilitation, even in the posterior areas.

## 5. Conclusions

Digital technologies allowed us to successfully resolve a challenging aesthetic case using zirconia, with a long-term follow-up of seven years. A long-practice approach with this new therapeutic option, along with deep knowledge of aesthetic ceramics, is recommended to manage the entailed drawbacks and limitations.

## Figures and Tables

**Table 1:** Therapeutic options proposed to the patient.

(a) Surgical removal of the deciduous tooth and orthodontic extrusion, and taking in occlusion of the permanent canine
(b) Conservative treatment of the deciduous tooth living in situ with the impacted canine
(c) Removal of the deciduous tooth and involvement of the adjacent natural elements for a fixed conventional or adhesive bridge living in situ with the impacted canine
(d) Removal of the deciduous tooth and replacement with implant-borne-prosthesis living in situ with the impacted canine, accepting the compromise of involving the impacted tooth in the implant seating procedure
(e) Surgical removal of the impacted canine and, in a second stage, removal of the deciduous tooth and replacement with implant-borne-prosthesis

## Data Availability

Not applicable.
